# Desire to Weigh Less and Unhealthy Weight Control Behaviors Among U.S. Adults: Evidence From NHANES 2017–2018

**DOI:** 10.1002/osp4.70177

**Published:** 2026-07-23

**Authors:** Anna V. Garcia, Elizabeth Dodge, Basil H. Aboul‐Enein

**Affiliations:** ^1^ College of Professional Studies, Applied Nutrition Graduate Program University of New England Portland Maine USA; ^2^ College of Arts & Sciences, Health & Society Program University of Massachusetts Dartmouth North Dartmouth Massachusetts USA; ^3^ Faculty of Public Health and Policy London School of Hygiene & Tropical Medicine London UK

**Keywords:** behaviors, disorders, NHANES, weight loss

## Abstract

**Background:**

With obesity rates remaining high in the United States, many individuals attempt weight loss using self‐directed approaches that may not align with clinical recommendations.

**Objective:**

This secondary data analysis examines the association between a desire to weigh less and unhealthy weight control behaviors among American adults.

**Methods:**

Data from the 2017–2018 National Health and Nutrition Examination Survey (NHANES) were assessed through variables representing a desire to weigh less and self‐regulated weight control behaviors that are not clinically recommended: skipping meals, using non‐RX weight loss supplements, and using laxatives or self‐induced vomiting. Chi‐square tests and a linear probability model were employed to evaluate their relationship.

**Results:**

Of adult survey respondents, 62.6% indicated a desire to weigh less. It was more likely to be reported by female, non‐Hispanic white, and college‐educated participants. Respondents who wanted to weigh less were more likely to engage in unhealthy weight loss strategies than those who wanted to gain weight or maintain their weight. There was a statistically significant relationship between a desire to weigh less and each unhealthy weight loss behavior (all *p* < 0.001). The linear probability model demonstrated that wanting to weigh less had the strongest effect on skipping meals.

**Conclusion:**

Findings indicate a strong relationship between a desire to lose weight and engaging in potentially harmful weight control behaviors, broadening our understanding of self‐regulated approaches that adults may resort to. More attention should be directed to patient weight satisfaction and monitoring of unhealthy weight control practices in patient‐provider collaborations through nutrition support and healthcare settings for improved weight management care.

## 1 Introduction

According to Centers for Disease Control and Prevention (CDC) data, approximately half of American adults report attempting to lose weight each year [1]. In the United States, adult obesity prevalence has reached 41.9% (measured from 1999–2022), while the prevalence of severe obesity in the United States (BMI ≥ 40 kg/m^2^) has more than doubled during the same period, increasing from 4.7% to 9.2% [2, 3], reflecting a combination of complex behavioral, environmental, and physiological factors influencing weight‐related practices [4]. Despite widespread recognition of obesity as a public health concern and broad individual motivation to achieve weight loss, awareness of the documented adverse health consequences of certain weight control methods remains inadequately understood at both the individual and population levels [5, 6].

Although short‐term weight loss may be achieved using a range of strategies, long‐term weight maintenance remains one of the most challenging aspects of obesity management for many individuals [7]. Evidence from decades of clinical research indicates that sustainable weight management is most effectively achieved through long‐term lifestyle modifications, particularly dietary changes that create a sustained negative energy balance and regular engagement in physical activity [8, 9, 10]. In addition, environmental influences, social norms related to body image, and the structure of the weight loss industry may contribute to individuals' desire to lose weight [6]. While individuals who report a desire to weigh less may seek different strategies for weight loss, not all approaches are clinically recommended; indeed, physiological responses to weight loss, including adaptive reductions in resting metabolic rate and alterations in appetite‐regulating hormones such as ghrelin, leptin, and peptide YY, may create a biological environment that predisposes individuals to weight regain [7, 11]. As a result, many individuals who succeed in short‐term weight loss struggle to maintain those reductions over time, and the majority of lost weight is typically regained within three to 5 years in the absence of sustained behavioral change [12, 13]. The multiple factors discussed above, including obesogenic environments, physiological processes, and food behavior, all can contribute to weight gain, and may in part explain why many individuals, despite genuine motivation to lose weight, turn to strategies that promise rapid results, even when such strategies are clinically unsupported or potentially harmful [14].

The decision to pursue weight loss and the specific strategies chosen are substantially shaped by environmental, cultural, and social forces. Societal norms that equate thinness with health, attractiveness, and personal discipline, often described as “diet culture”, create powerful incentives to pursue rapid or dramatic weight loss over sustainable behavioral approaches [6, 15]. Research has demonstrated that exposure to appearance‐focused social media content is associated with increased body dissatisfaction, decreased body satisfaction/appreciation, increased fear of negative assumptions based on appearance, and more frequent engagement in disordered eating behaviors [16]. Body dissatisfaction, in turn, is a well‐established predictor of the adoption of unhealthy weight control practices, including extreme and restrictive dieting, meal skipping, and purging behaviors [17, 18, 19, 20]. Although no consensus has been established among scientists, disordered eating is often described as the presence of typical symptoms of eating disorders that occur less frequently or with less seriousness than those of full‐blown eating disorders [21]. While some patterns of disordered eating are not recognized in the current Diagnostic and Statistical Manual of Mental Disorders, Avoidant/Restrictive Food Intake Disorder has been added to the most recent version, and may result in increased diagnoses of previously uncategorized or under‐diagnosed eating disorders, including those related to self‐regulation and the desire to see short‐term results in weight loss [22, 23, 24]. Some of these self‐regulated strategies include skipping meals, using non‐prescription supplements, and using laxatives or self‐induced vomiting [2, 18, 25]. For many, the preferred methods for weight management are short‐term strategies that are often accomplished quickly, accompanied by a willingness to look past the potential health risks of such unhealthy weight control practices.

The broader structural consequence of repeated, short‐term weight loss attempts without sustainable behavioral change is weight cycling, a pattern of intentional weight loss followed by unintentional weight regain repeated across multiple episodes [14, 26]. Weight cycling is not merely a benign marker of unsuccessful dieting; it has been associated with a range of serious long‐term health consequences. Compared to weight stability, weight cycling has been associated with an approximately 30% increased risk of obstructive sleep apnea, and type 2 diabetes, and with a greater than 50% increased risk of heart failure [2, 14, 27]. Engaging in unhealthy weight loss strategies also puts the individual at a greater risk of developing full‐blown eating disorders, defined as a continuous disturbance of eating or eating‐related behavior that is harmful to health or psychosocial functioning [12, 13, 26, 28]. For example, self‐induced vomiting and the use of laxatives following uncontrolled episodes of overeating to prevent weight gain are associated with bulimia nervosa [29] Losing weight can pose important challenges on its own, but keeping lost weight off has been proven to be even more difficult [30, 31]. Evidence suggests that sustainable weight management is typically achieved through long‐term lifestyle modifications such as dietary changes and regular physical activity [31], and that short‐term weight loss solutions may be both psychologically and physiologically harmful.

Despite the clinical significance of unhealthy weight control behaviors and their established associations with disordered eating and cardiometabolic harm, the specific association between a desire to weigh less, as a motivational antecedent, and the adoption of these practices among U.S. adults remains incompletely characterized in large, nationally representative samples. Much of the existing literature has focused on adolescent or clinical eating disorder populations, or has examined unhealthy weight control behaviors in aggregate rather than in direct relation to weight loss motivation, limiting the generalizability of findings to the broader adult population [5, 6]. Furthermore, few studies have simultaneously examined multiple categories of unhealthy weight control behavior, meal skipping, non‐prescription supplement use, and laxative use or self‐induced vomiting, within the same analytical framework, preventing a comprehensive understanding of how the desire to lose weight maps onto this spectrum of harmful practices. Therefore, the primary outcome of this study is to explore whether there is an association between a desire to weigh less and unhealthy weight loss strategies, including skipping meals, using non‐RX supplements, and vomiting or using laxatives for weight loss among U.S. adults. The research will utilize National Health and Nutrition Examination Survey (NHANES) data to conduct a secondary data analysis. In addition to addressing the core problems mentioned, it can be utilized to build upon existing assumptions of healthy approaches to weight loss and to propose new evidence‐based alternatives that nutrition professionals can recommend to patients currently engaging in unhealthy weight loss methods.

## 2 Methods

### 2.1 Data Source

The data for this secondary data analysis were downloaded from the National Health and Nutrition Examination Survey (NHANES) 2017–2018 dataset. NHANES is a cross‐sectional observational study that assesses the health and nutritional status of Americans to make generalized estimates, taking into account diseases and risk factors for health; the survey is conducted in all 50 states and Washington, D.C. with approximately 5000 participants each year [32], providing one of the few nationally representative datasets large enough to examine these behaviors reliably in adults. An introduction to NHANES and the detailed data collection methods for the dataset in question can be obtained from the official website [33].

The 2017–2018 NHANES cycle was selected because it represents the most recent fully completed pre‐pandemic dataset.

This secondary data analysis used publicly available, de‐identified data and qualifies as exempt from institutional review board (IRB) review under U.S. federal regulations (45 CFR 46.104(d)(4)). Institutional guidance notes that if the data set is publicly available and fully de‐identified, the use of the data would not meet the definition of research involving human subjects per 45 CFR 46, which was applied by the authors in declining to submit an IRB application for NHANES data.

From the multiple surveys that the NHANES consists of, this study analyzed data collected from the Demographics Data and Weight History Questionnaire, which includes participants aged 16 and older. The data was restricted to participants aged 21 and older to draw conclusions from an adult population sample, excluding children and adolescents.

### 2.2 Variables

#### 2.2.1 Demographic Characteristics

The following four variables were extracted from the Demographics Data and included as covariates in the analyses: age, gender (male/female), race (Mexican American, other Hispanic, Non‐Hispanic White, Non‐Hispanic Black, Non‐Hispanic Asian, and other), and education (less than ninth grade, 9–11th grade, high school graduate/GED or equivalent, some college or AA degree, and college graduate or above). Body mass index was intentionally excluded to avoid overadjustment and to maintain a clear interpretation of dissatisfaction‐driven behaviors.

#### 2.2.2 Independent Variable: Desire to Weigh Less

The dependent variable was constructed from a Weight History Questionnaire question that asked participants: “Would you like to weigh more, less, or the same?”. This variable had three response categories: “less”, “more”, and “stay about the same”. The “more” and “stay about the same” categories were combined to isolate a desire to weigh less. It was determined that this variable was the best available in this dataset to serve as a proxy for wanting to lose weight, as the “weigh more” and “stay the same” response categories both reflected the absence of wanting to lose weight, and therefore do not represent the construct of interest. Participants who reported a desire to gain weight were retained in the reference group to preserve the full analytic sample. The decision to dichotomize a desire to weigh less was theoretically justified and consistent with prior research that conceptualizes dissatisfaction as a binary indicator [34, 35]. Throughout this article, the terms “independent variable,” and “desire to weigh less” are used interchangeably to describe the same construct, with terminology varying based on statistical or conceptual context.

#### 2.2.3 Dependent Variables: Unhealthy Weight Loss Strategies

Using items from the Weight History Questionnaire, the following three behavioral variables serving as answers to the question “How did you try to lose weight?” were operationalized to represent weight loss strategies that are generally considered as unhealthy: (1) skipped meals; (2) use of other pills, medicines, herbs, or supplements not needing a prescription; and (3) use of laxatives or vomiting as behaviors that align with established classifications of unhealthy and extreme weight control practices in prior literature [36]. These variables had two response categories: “no” and “yes”. A small number of respondents stated “I don't know”, and these were combined with the “no” responses.

In the absence of formal guidelines for categorizing weight loss methods, Project EAT, a survey assessing a wide range of weight‐related problems among adolescents, has introduced an alternative for classification. According to this system, skipping meals is categorized as an “unhealthy weight control behavior”, whereas taking diet pills or supplements, making oneself vomit, and using laxatives all fall under “extreme weight control behaviors” [37]. It is important to emphasize that while there are many other weight loss behaviors that can be considered unhealthy, given the few items in the NHANES questionnaires addressing approaches to losing weight, the selected variables were considered the best available to represent them.

### 2.3 Data Management

All datasets were downloaded from the official NHANES website utilizing the 2017–2018 edition of the survey [33]. Once the NHANES Demographic Data and Weight History Questionnaire were downloaded, each dataset was imported and saved individually using Stata statistical software. The two datasets were merged using a common identifier and saved as one file. This resulted in 3093 cases that had demographic data but did not exist in the weight dataset. As such, the unmatched cases were dropped.

The analytical sample was restricted to participants aged 21 years and older to focus on an adult population and ensure consistency across variables. Then, all the variables that were irrelevant to the research question were cleared out, keeping only the demographic characteristics of age, gender, race and education, a desire to weigh less, and unhealthy weight control strategy variables of skipped meals, taking non‐RX weight loss supplements, and taking laxatives or vomiting. All variables and their values were labeled based on the NHANES codebook for the purpose of clarity and better record‐keeping. Finally, the cleaned dataset was saved in preparation for conducting statistical analyses.

### 2.4 Statistical Analyses

Two analytical approaches were employed in this research. Pearson's chi‐square test was used to assess associations between the desire to weigh less and each unhealthy weight control behavior. In preparation for the test, the dependent variables had to be converted into binary variables in Stata. The dependent variables were originally scored in such a way that only respondents who answered “yes” to the question were not coded as missing. That is, if a respondent did not report engaging in the behavior, they were not scored as “no”, but instead as a missing value. These missing values were recoded to “no” for the purposes of analysis. A limitation of this approach is that some who did not answer “yes” may have been individuals who did not know or refused to answer the question, but this cannot be differentiated from the current data. The predictor—the desire to weigh less—was also recoded from three categories into two for the purposes of the statistical tests. The desire to weigh more and maintain current weight were combined as categories to produce more intuitive results.

As a second statistical test, a regression analysis was conducted to help understand unhealthy weight loss behaviors and predict whether engaging in those behaviors was a result of a desire to weigh less. A linear probability model (see Table 1) was used to adjust for the four demographic characteristics described earlier. This model was used to ensure interpretable probability changes relevant to clinical and public health applications [38]. With NHANES's large sample, the model's limitations were minimized [39]. Results were expressed as regression coefficients and as *R*‐squared values with corresponding 95% confidence intervals and *p*‐values.

**TABLE 1:** Linear probability model to predict each behavior by the desire to weigh less, controlling for demographics.

	Skipping meals	Taking non‐RX supplements	Taking laxatives or vomiting
	*b* (se)		
Desire to weigh less	0.111^***^	0.025^***^	0.009^***^
	(0.008)	(0.004)	(0.002)
Education level			
9–11th grade	0.024	0.012	0.004
	(0.018)	(0.010)	(0.005)
High school/GED	0.023	0.019^*^	0.006
	(0.017)	(0.009)	(0.005)
Some college/AA	0.027	0.025**	0.001
	(0.017)	(0.009)	(0.005)
College grad or above	0.023	0.017	−0.000
	(0.017)	(0.009)	(0.005)
Race			
Other Hispanic	−0.011	−0.006	0.003
	(0.017)	(0.009)	(0.005)
Non‐Hispanic white	−0.034^*^	−0.018^*^	−0.003
	(0.014)	(0.007)	(0.004)
Non‐Hispanic black	0.011	−0.010	0.012^**^
	(0.015)	(0.008)	(0.004)
Non‐Hispanic Asian	−0.029	−0.031^***^	−0.001
	(0.016)	(0.008)	(0.004)
Other	0.028	0.012	0.003
	(0.021)	(0.011)	(0.006)
Age	−0.001^**^	−0.001***	−0.000
	(0.000)	(0.000)	(0.000)
Gender	−0.015	0.007	0.002
	(0.008)	(0.004)	(0.002)
Intercept	0.077^**^	0.020	−0.001
	(0.024)	(0.012)	(0.006)
R2	0.0393519	0.0197094	0.0098414
Number of observations	5475	5475	5475

*Note:* Coefficients with standard errors in parentheses.

^*^ *p* < 0.05.

^**^ *p* < 0.01.

^***^ *p* < 0.001.

To estimate the minimum sample size needed to find a statistically significant effect of the independent variable on each dependent variable, three post hoc power analyses were conducted (see Table 2) in Stata based on the coefficient, standard error, standard deviation, power, and alpha levels from the regression model. An alpha level of 0.01 was determined to be appropriate for the calculation given the large sample size of the NHANES survey. All analyses were conducted using version 18.0 of Stata/BE statistical software [40].

**TABLE 2:** Post hoc power analysis.

Disordered weight control behavior	Required sample size
Skipping meals	5
Taking non‐RX supplements for weight loss	7
Taking laxatives or vomiting	8

*Note:* Power calculated at *α* = 0.01, power = 0.90. Estimates derived from the regression results in Table 1.

Analyses were conducted without the application of NHANES sampling weights. While weights are required for generating nationally representative prevalence estimates, their use is not universally required when the primary objective is to estimate associations between variables rather than population‐level parameters. Prior methodological research indicates that unweighted regression models can provide valid estimates of associations when the objective is relational rather than descriptive and when models adjust for key covariates [41, 42, 43].

## 3 Results

**FIGURE 1:**
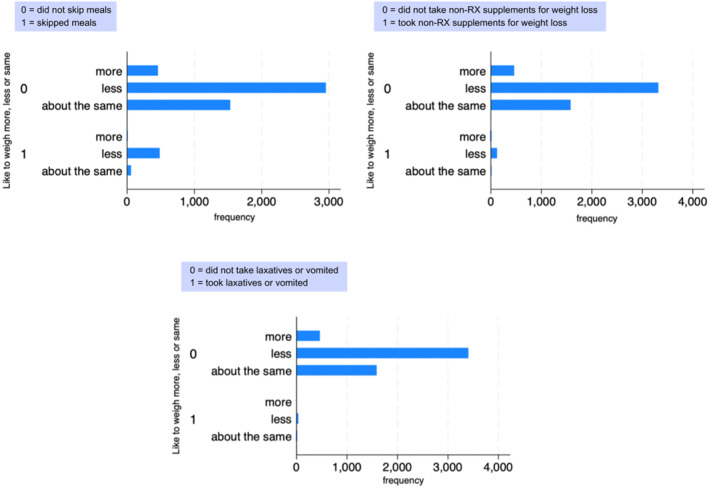
Frequencies of disordered weight loss behaviors by weight satisfaction and dissatisfaction.

The results demonstrated that 62.6% of adult survey respondents over 21 years of age indicated having a desire to weigh less compared to a desire to weigh more (8.4%) or to maintain current weight (28.9%). As can be observed in Table 3, respondents who desired to weigh less were more likely to be female (58.2% vs. 41.8% respectively), and more likely to be non‐Hispanic White (36.7%) as opposed to other racial or ethnic groups, compared to respondents who wanted to weigh more or were satisfied with their current weight. Most individuals who desired to weigh less had some college education (34.6%) or were college graduates or above (26%). The average age across all weight satisfaction groups was relatively similar; however, those desiring to weigh more were slightly younger. The distribution of unhealthy weight control behaviors against the desire to weigh less is illustrated in Figure 1. These findings suggest that the examined behaviors were relatively infrequent in the general population.

**TABLE 3:** Characteristics of NHANES survey participants by weight satisfaction status.

	Desire to weigh more *n* (%)	Desire to weigh less *n* (%)	Desire to maintain weight *n* (%)	Don't know *n* (%)	Total *n* (%)
*N*	462 (8.4)	3437 (62.6)	1589 (28.9)	5 (0.1)	5493
Gender					
Male	299 (64.7)	1438 (41.8)	925 (58.2)	2 (40.0)	2664 (48.5)
Female	163 (35.3)	1999 (58.2)	664 (41.8)	3 (60.0)	2829 (51.5)
Race					
Mexican American	38 (8.2)	485 (14.1)	194 (12.2)	1 (20.0)	718 (13.1)
Other Hispanic	30 (6.5)	330 (9.6)	148 (9.3)	0	508 (9.2)
Non‐Hispanic white	146 (31.6)	1260 (36.7)	505 (31.8)	2 (40.0)	508 (9.2)
Non‐Hispanic black	161 (34.8)	755 (22.0)	365 (23.0)	0	1281 (23.3)
Non‐Hispanic Asian	58 (12.6)	440 (12.8)	302 (19.0)	2 (40.0)	802 (14.6)
Other	29 (6.3)	167 (4.9)	75 (4.7)	0	271 (4.9)
Education level					
Less than high school	35 (7.6)	250 (7.3)	192 (12.1)	1 (20.0)	478 (8.7)
9–11th grade	77 (16.7)	337 (9.8)	209 (13.2)	2 (40.0)	625 (11.4)
High school/GED	131 (28.4)	765 (22.3)	395 (24.9)	1 (20.0)	1292 (23.5)
Some college/AA	151 (32.7)	1189 (34.6)	408 (25.7)	1 (20.0)	1749 (31.8)
College grad or above	67 (14.5)	892 (26.0)	377 (23.7)	0	1336 (24.3)

	**Mean (sd)**	**Mean (sd)**	**Mean (sd)**	**Mean (sd)**	**Mean (sd)**
Age	47.136 (19.524)	51.162 (16.532)	55.009 (18.527)	54.400 (25.851)	51.940 (17.543)

The Pearson's chi‐square tests indicated that respondents who wanted to weigh less were more likely to engage in unhealthy weight loss strategies than respondents who desired to gain weight or maintain their weight. There was a statistically significant relationship between individuals' desire to weigh less, and the behaviors of skipping meals (*p* < 0.001, *χ* ^2^ = 173.52), taking non‐RX supplements to lose weight (*p* < 0.001, *χ* ^2^ = 46.80), and taking laxatives or vomiting (*p* < 0.001, *χ* ^2^ = 15.67) compared to a desire to weigh more or maintain current weight.

We estimated a linear probability model to control for the effect of demographic variables on unhealthy weight control behaviors. This model demonstrated that the coefficient for the desire to weigh less was largest for skipping meals relative to the other behaviors, indicating that the desire to weigh less had a stronger effect on skipping meals. Although the coefficients for taking laxatives or vomiting for weight loss and taking non‐RX supplements for weight loss were also statistically significant, increasing the probability of engaging in the behavior by 0.009 and 0.025, respectively, they were rather small in practical terms.

Finally, a post hoc power analysis to determine the power of the study revealed that the minimum sample size required for statistical significance was much lower than the actual sample size, which indicated that the utilized dataset was indeed sufficiently large to produce statistically significant results.

## 4 Discussion

The findings of this study indicate a strong relationship between a desire to weigh less and engaging in less‐than‐ideal weight control behaviors among adults, broadening our understanding of self‐regulated approaches that individuals who attempt to lose weight may resort to.

This study fills two important gaps in existing research. Firstly, the focus of most literature on both unhealthy weight loss behaviors and their associations, as well as individuals' dissatisfaction with weight and body image has been placed on adolescents. Surprisingly, little is known about unhealthy weight control strategies when it comes to the U.S. adult population, although adult‐specific data is frequently collected and readily available through a nationally conducted survey. Secondly, most nutrition research focuses on the physical determinants of body weight, creating another notable gap in research involving the assessment of psychological factors and how they may impact self‐monitored weight control practices.

Given that an association between a desire to weigh less and unhealthy weight loss strategies was established as an outcome of the research, this can increase nutrition professionals' comprehension of the challenges that patients commonly face, facilitating improved screening practices and public health messaging. Given the strong association between seeking weight loss and unhealthy weight control behaviors among U.S. adults, public health practitioners should consider weight dissatisfaction as a potential early indicator of engagement in harmful weight‐related practices. This helps pave the way for much‐needed empathetic new approaches and patient‐provider collaborations. The results demonstrate that more attention should be directed on patient weight dissatisfaction and monitoring of unhealthy weight control practices in patient‐provider collaborations through nutrition support and healthcare settings for improved weight management care. As such, the findings highlight a need for improved screening for weight dissatisfaction and harmful behaviors.

Furthermore, the results can contribute to preventing unhealthy weight loss practices from transforming into eating disorders. With a wide array of potential benefits, this study plays a fundamental role in strengthening future approaches of obesity care and prevention programs that encourage healthy, sustainable weight management practices, allowing both nutrition professionals and individuals seeking to lose weight to benefit from the results of this study. This research is designed precisely to assist with the development of more effective, compassionate long‐term weight management interventions, which are greatly needed to combat the complex series of processes implicated in the obesity epidemic. The framing in this study emphasizes that dissatisfaction often arises from cultural norms, environmental pressures, and the biological difficulty of sustained weight loss, rather than from personal shortcomings.

Nevertheless, the findings should be considered in light of certain limitations. Perhaps most importantly, as a secondary data analysis, there are existing limitations with the NHANES data variables with few questionnaire items addressing approaches to losing weight. All behaviors were self‐reported and may be subject to recall or social desirability bias. NHANES demographics data, such as education level, grouped individuals between 6 and 19 years of age in the children/youth variable, whereas individuals aged 20 and older were included in the adult variable, posing a challenge for data collection and another motive for setting the age limit of this study at 21. In addition, income level was initially considered as a fifth covariate for the study, but we later excluded it because the income level categories not being mutually exclusive per the NHANES codebook. Future studies may benefit from exploring other publicly available dataset alternatives to investigate the desire to lose weight and unhealthy weight control practices through a larger variety of variables relevant to weight management strategies.

The use of a single NHANES cycle may limit comparability across time; however, this decision was made to avoid introducing heterogeneity associated with pandemic‐related disruptions. Combining NHANES cycles was considered but not pursued due to differences in data completeness and analytic guidance from the National Center for Health Statistics (NCHS), which cautions against combining incomplete or methodologically inconsistent cycles, particularly those affected by the COVID‐19 pandemic. Data collection was disrupted beginning in 2019–2020 due to the pandemic, resulting in incomplete cycles that cannot be combined using standard analytic procedures. Pandemic‐era data introduce major contextual confounders related to stress, healthcare access, food insecurity, and body weight changes that would meaningfully alter interpretation. Given these considerations and because this study aimed to examine associations under stable population conditions, restricting the analysis to 2017–2018 ensured methodological consistency and interpretability [44, 45].

Although the findings indicate that non‐Hispanic White individuals were more likely to report a desire to lose weight, elaboration on sociocultural explanations regarding was beyond the scope of this methods‐driven, data‐focused secondary analysis. The purpose of the study was to quantify associations rather than to speculate on causal sociocultural mechanisms that cannot be empirically evaluated using the available NHANES data. Expanding such interpretations without direct measures would extend beyond the evidence and risk unsupported inference; however, this represents a potentially valuable direction for future research. It should also be noted that the objective of the study is to assess associations and not to construct a full predictive model of weight dissatisfaction or behaviors.

Another limitation of this study is that NHANES sampling weights were not applied. As a result, the findings are not intended to produce nationally representative prevalence estimates. However, because the primary objective was to assess associations between variables, and models adjusted for key demographic covariates, the impact on estimated relationships is expected to be limited. Future research may extend these findings using weighted analyses to assess population‐level generalizability.

While the inclusion of post hoc power estimates serves a practical purpose in secondary analyses of NHANES data, namely to demonstrate that the sample provides adequate sensitivity to detect effects at the population level, there is ongoing debate regarding the interpretive value of post hoc power [46]. Despite this debate, post hoc power reporting remains a standard practice in epidemiologic and health science literature, particularly in studies where effect sizes are small but statistically significant [47]. In this context, post hoc power does not bias interpretation; rather, it enhances transparency by clarifying the magnitude of effects that the analysis was sufficiently powered to detect.

The R‐squared values reported with the results of the study indicate that much of the variation in behaviors related to self‐managed weight control strategies is still left unexplained. This is particularly accurate for taking laxatives or vomiting for weight loss, as the relevant data to this behavior is limited with only 36 survey respondents reporting engaging in it. Given the sensitive nature of discussing unideal weight control practices in a public manner, the setting of NHANES interviews may not be exemplary for participants to reach a level of comfort needed to admit to engaging in these strategies as part of an extensive survey that collects data on a variety of health topics.

## 5 Conclusion

In conclusion, these strengths and limitations indicate a critical need for further research on the topic of this study. Researchers should focus their efforts on investigating the explored paradigms in more depth, ideally with the inclusion of a qualitative approach conducted through semi‐structured interviews. This can help create a more intimate environment for participants to share their experiences on unhealthy weight control practices and weight dissatisfaction, and that way, for the investigators to gain a more comprehensive and well‐rounded understanding of the challenges they face.

## Author Contributions

**Anna V. Garcia:** conceptualization, data curation, methodology, formal analysis, project administration, resources, software, supervision, writing – original draft, writing – review and editing. **Elizabeth Dodge:** project administration, resources, supervision, writing – original draft, writing – review and editing. **Basil H. Aboul‐Enein:** writing – review and editing.

## Funding

The authors have nothing to report.

## Ethics Statement

The authors have nothing to report.

## Conflicts of Interest

The authors declare no conflicts of interest.

## Data Availability

Data are derived from public domain resources.
